# High Power Thermoelectric Generator Based on Vertical Silicon Nanowires

**DOI:** 10.1021/acs.nanolett.0c00227

**Published:** 2020-05-28

**Authors:** Shaimaa Elyamny, Elisabetta Dimaggio, Stefano Magagna, Dario Narducci, Giovanni Pennelli

**Affiliations:** † Dipartimento di Ingegneria della Informazione, Università di Pisa, Via G. Caruso, I-56122 Pisa, Italy; ‡ Electronic Materials Research Department, Advanced Technology and New Materials Research Institute, City of Scientific Research and Technological Applications (SRTA-City), New Borg El-Arab City, 21934, Alexandria, Egypt; § Department of Materials Science, University of Milano Bicocca, via R. Cozzi 55, 20125 Milan, Italy

**Keywords:** thermoelectricity, silicon nanowires, thermal conductivity, power density

## Abstract

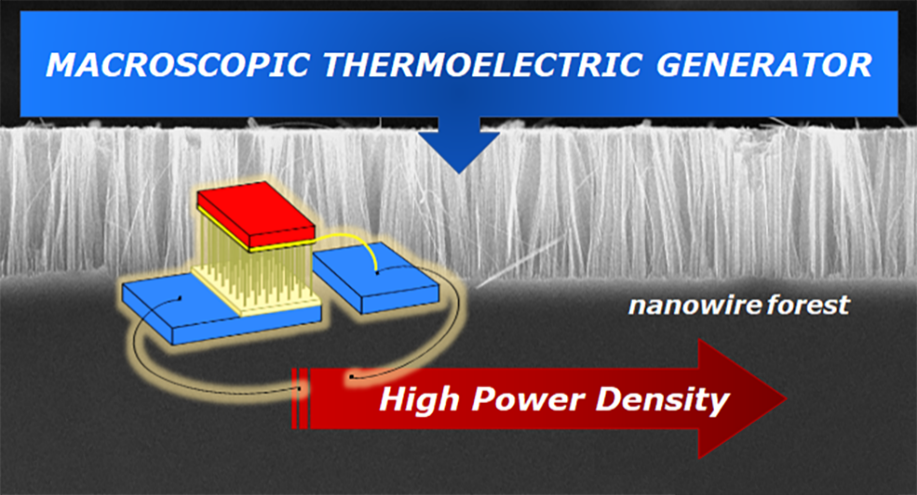

Thermoelectric generators, which convert heat directly into electrical power, have great potentialities in the energy harvesting field. The exploitation of these potentialities is limited by the materials currently used, characterized by good thermoelectric properties, but also by several drawbacks. This work presents a silicon-based thermoelectric generator, made of a large collection of heavily *p*-doped silicon nanostructures. This macroscopic device (area of several mm^2^) collects together the good thermoelectric features of silicon, in terms of high power factor, and a very reduced thermal conductivity, which resulted in being exceptionally low (1.8 W/(m K), close to the amorphous limit). The generated electrical power density is remarkably high for a Si-based thermoelectric generator, and it is suitable for scavenging applications which can exploit small temperature differences. A full characterization of the device (Seebeck coefficient, thermal conductivity, maximum power output) is reported and discussed.

## Introduction

Nanostructured silicon is a very good thermoelectric material.^1^ Its Seebeck coefficient *S* and electrical conductivity σ can be tailored by doping,^2,3^ so that its power factor *S*^2^σ can reach values in excess of 5 mW/(m K^2^) at room temperature. Additionally, nanostructuring offers a via for the reduction of the phonon propagation,^4,5^ and hence of the thermal conductivity *k*_t_. A strong reduction of *k*_t_ in silicon nanowires has been experimentally demonstrated by several groups.^6−12^

Besides the excellent thermoelectric properties (when nanostructured), silicon is a low cost and very sustainable material, and moreover, its physical and technical properties are very well-known for its pervasiveness in the electronic market. Hence, the use of silicon for direct thermal to electrical energy conversion will be disruptive for a large range of scavenging and green energy harvesting applications, which are currently limited by the available thermoelectric devices based on rare and non-environmentally friendly materials. The potentialities of silicon as a thermoelectric material have been assessed with devices based on one (or very few) nanowires.^11,13,14^ However, the crucial point to be addressed for practical thermoelectric applications of silicon is to combine large amounts of nanostructures with suitable electrical and thermal connections, so that macroscopic thermoelectric generators (TEGs) capable of delivering enough electrical power can be produced. Large collections of silicon nanostructures can be achieved by bottom-up approaches, based on chemical-vapor deposition (CVD) through the vapor liquid solid (VLS) mechanism.^15,16^ In this context, interesting and very promising solutions to assemble VLS nanowires with suspended microelectromechanical (MEMS) Si platforms have been developed.^17−19^ Large arrays of interconnected nanowires can be fabricated also by top-down approaches, based on complex processes, which involve high resolution lithography and etching.^20,21^ Vertical arrays of nanowires have been produced both by deep-reactive ion etching (DRIE)^22,23^ and by VLS.^24^

**Figure 1:**
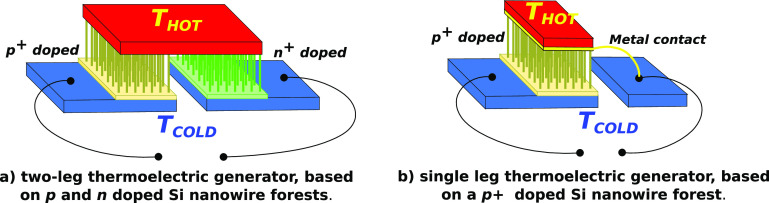
Sketches of thermoelectric generators, based on silicon nanowire forests.

We manufactured and characterized macroscopic TEGs of several mm^2^ of surface, based on vertical Si nanowires achieved by the inexpensive and rather simple metal assisted chemical etching (MACE) technique. Figure 1 shows sketches of a thermoelectric module that uses large collections of interconnected vertical silicon nanowires (SiNW forests). The ideal case would be to have SiNW forests both *p*^+^- and *n*^+^-doped, interconnected as shown in the sketch of Figure 1a. As it is very difficult to achieve nonporous nanowires on heavily *n*-doped substrates,^25,26^ we fabricated and fully characterized single-leg TEGs modules based on *p*^+^ nanowires (see the sketch of Figure 1b).

In this article, after resuming the fabrication process, from MACE to the assembly of the module, we report a full thermal and electrical characterization, including the Seebeck coefficient and the thermal conductivity which resulted in being smaller than 2 W/(m K). Even more remarkable, the measured electrical power output density resulted in being very high for thermoelectric silicon devices and comparable with that of commercially available TEGs.

## Results

### Low Cost Arrays of Long Nanowires on Large Si Areas

**Figure 2:**
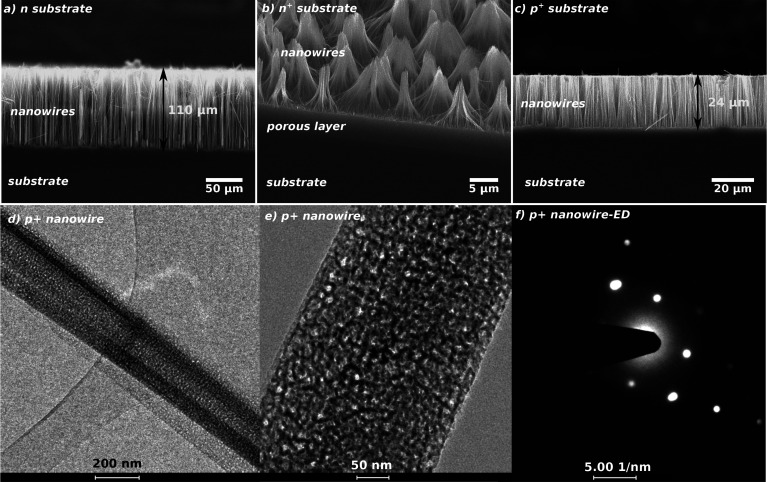
In the top panels: cross-section SEM images of silicon nanowire forests, fabricated on Si substrates with different doping concentrations: (a) *n*-doped, (b) *n*^+^-doped, (c) *p*^+^-doped. In the bottom panels: TEM images (d, e) and electron diffraction (ED) analysis (f) of a *p*^+^ nanowire.

The metal assisted chemical etching^27,28^ (MACE) offers the opportunity for the affordable production of a large amount of vertical silicon nanowires with high length-to-diameter aspect ratio. It consists of soaking a silicon substrate (wafer) in a solution containing hydrofluoric acid (HF) and a metal salt,^29−32^ such as silver nitrate (AgNO_3_). Even if, for our purposes, samples with a surface of roughly 1 × 1 cm^2^ have been fabricated, the technique can be applied to larger surfaces to produce forests of nanowires placed perpendicularly to the initial silicon substrate. We applied the MACE process on silicon substrates (wafers) with different doping (see Figure 2): slightly *n*- and *p*-doped (resistivity 1–10 Ω·cm), moderately *n*-doped (resistivity 0.5–1 Ω·cm), and heavily *n*^+^- and *p*^+^-doped (resistivity 0.003–0.005 Ω·cm). We found that MACE is very reliable on substrates with a doping concentration smaller than 10^18^ cm^–3^, both *n* and *p* type: the length of the nanowires is limited only by the etching time and by the volume of the solution. Figure 2a shows a cross-section SEM image of a typical nanowire forest achieved by etching a *n* substrate for 8 h at 18 °C (see the Supporting Information for more details on the parameters and on the etching procedure): nanowires are 110 μm long with an average diameter of 80 nm. As high doping concentrations are required for the maximization of the power factor^33^*S*^2^σ, nanowires need to be doped by thermal diffusion after their fabrication.^3,34^ However, the substrate remains undoped, resulting in a high parasitic electrical resistance in series with the nanowires which reduces the funcionality of the thermoelectric generator. A possible solution would be to implement complex techniques for the removal of the substrate and for the mechanical stabilization of the nanowires.

As an alternative, a simple solution for the reduction of the parasitic resistance would be to fabricate nanowires directly on heavily doped substrates. To this end, we experimented with a wide range of etching parameters in order to achieve nanowire forests on heavily doped *n*^+^ and *p*^+^ substrates. Figure 2b shows a typical result achieved with a *n*^+^ substrate: a thick layer of porous silicon is visible in the substrate under the nanowires. We tested several HF/AgNO_3_ concentrations, temperatures, and etching times,^26^ and we always achieved porous nanostructures on *n*^+^-doped substrates. More details on the effect of reagent concentrations and etching temperature are given in the Supporting Information. Porous structures are not optimal for thermoelectric generation. Indeed, from one side, the thermal conduction is reduced because the porosity increases the phonon scattering, and this is beneficial for thermoelectric purposes; from the other side, also the electrical conductivity σ decreases because the porosity affects the electron scattering as well. Moreover, the cross section available for the electrical conduction of porous nanowires is smaller with respect to that of monocrystalline nanowires, and this causes a reduction of the deliverable current. The ideal case would be to have monocrystalline, heavily doped nanowires, where the phonon transport is reduced by the surface scattering; meanwhile, the electron (or hole) transport is the same as the bulk silicon: the electrical conductivity in heavily doped silicon is only slightly affected by the surface scattering for nanowires larger than 40 nm.^35^

In the case of *p*^+^ substrates, we found a suitable combination of reagent concentrations and etching temperatures, which can result in nanowire forests without a porous layer at the bottom. Figure 2c shows a nanowire forest fabricated by MACE on a *p*^+^ substrate in a solution of HF:AgNO_3_:H_2_O 3:16:60 for 3 h at 18 °C. The length of the nanowires is of 24 μm. No porosity is visible at the bottom of the nanowires. The morphology of the *p*^+^ nanowires has been further investigated through a transmission electron microscope (TEM). The inspections have been performed on single nanowires detached from the nanowire forest. Chips were immersed in IPA and treated in ultrasonic bath for 5 min, and then, drops of the solution containing the nanowires were placed on carbon holey grids. After the evaporation of the solvent, nanowires were ready for image acquisitions. The TEM images and electron diffraction (ED) analyses were acquired using a LaB6 FEI Tecnai TEM at 200 kV, and the results are visible in the bottom panels of Figure 2. Parts d and e of Figure 2 clearly show the porous and rough surface of the single nanowire; nonetheless, the electron diffraction analysis shown in Figure 2f, performed on the same nanowire, highlights a diffraction pattern typical of monocrystalline silicon, which is the core of the *p*^+^ nanowire. Hence, silicon nanowire forests fabricated on *p*^+^ substrates exhibit a monocrystalline core, porous surfaces for the reduction of the phonon scattering, and a highly doped substrate with a reduced parasitic resistance.

After the fabrication of the *p*^+^ nanowire forest, a single-leg thermoelectric generator can be easily made: the top of the nanowires can be contacted by means of copper electrodeposition, following a process reported in previous works.^34^ The process for the fabrication of the contact on the top of the SiNW forest does not require any filling material,^22−24^ which would introduce an unwished parallel thermal conduction. The silicon substrate at the bottom acts as a good contact, for both the electrical and the thermal transport, and gives also a good mechanical stability to the structure.

### Thermal and Electrical Characterization

**Figure 3:**
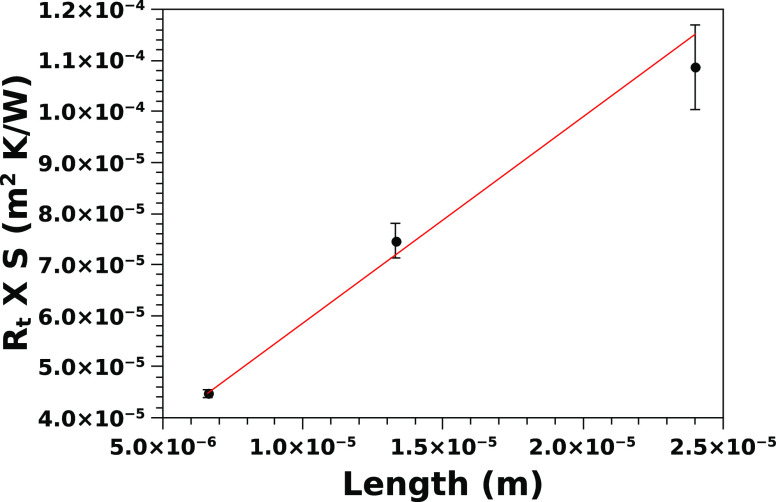
Thermal resistance, multiplied by the surface, as a function of the nanowire length. The slope of the linear fit is the reciprocal of the thermal conductivity *k*_t_.

In a previous work,^36^ we measured the thermal conductivity of large forests of Si nanowires fabricated on low *n*-doped substrates (resistivity 1–10 Ω cm, doping concentration 10^15^ cm^–3^). The thermal conductivity resulted in being 4.6 W/(m K), which is very small with respect to that of bulk silicon (148 W/(m K)). As low doped nanowires are unsuitable for thermoelectric purposes, in this work, we focus on *p*^+^ nanowires. The measurement of the nanowire thermal conductivity has been performed with the guarded hot plate technique previously developed.^36^Figure 3 reports the thermal resistance of *p*^+^ SiNW forests of different length, multiplied by the surface of each sample. The linear fit is also reported on the graph: the slope is the reciprocal of the thermal conductivity multiplied by the filling factor ν, which is the ratio between the real surface of the nanowires and the overall surface of the samples.^36^ From the slope, we achieved *νk*_t_ = 0.25 ± 0.02 W/(m K). From SEM top-views of the samples, ν has been estimated to be ν = 0.14 ± 0.01 (see the Supporting Information for a description of the measurement procedure for ν), and hence, the thermal conductivity resulted in being *k*_t_ = 1.8 ± 0.3 W/(m K). The intercept with the vertical axis of the linear fit, shown in Figure 3, is the thermal resistance of the contacts, which resulted in being 1.814 × 10^–5^ (m^2^ K)/W.

**Figure 4:**
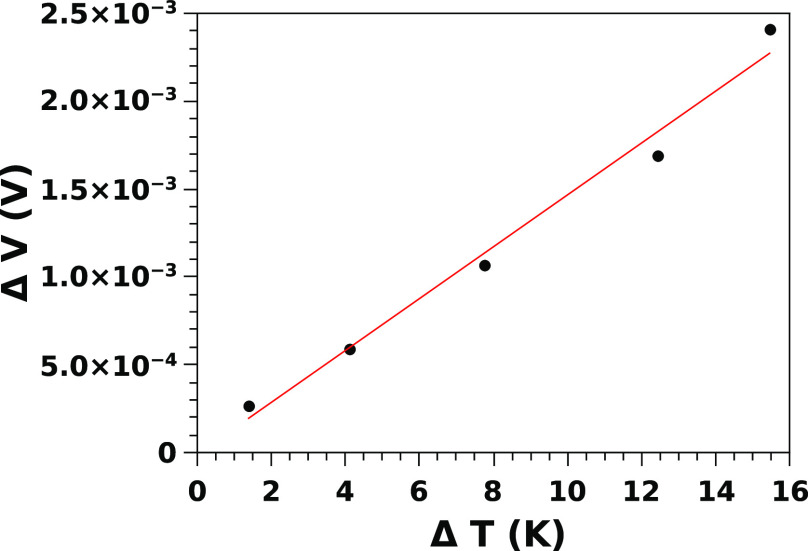
Seebeck voltage as a function of the temperature difference, evaluated from the top-to-bottom total temperature difference minus the temperature drop due to the thermal contact resistances.

The Seebeck coefficient of *p*^+^-doped SiNW forests has been measured simultaneously with the thermal conductivity. The temperature difference has been recorded between the heated top plate and the cooled bottom plate, and the output voltage drop has been recorded by means of a nanovoltmeter (Keithley 2182). The temperature drop on the contacts has been evaluated knowing the heat flux and the contact thermal resistance, measured as explained previously. The Seebeck voltage is due to the effective temperature difference between the ends of the nanowires, which has been determined subtracting the temperature drop on the contacts from the total measured temperature difference. The temperature drop of the substrate has been considered negligible, as also the Seebeck voltage of the copper wires used for the voltage measurements. Figure 4 shows the Seebeck voltage as a function of the effective temperature drop between the ends of the nanowires for the 2 h SiNW forest (*L* = 13.5 μm). The linear fit of this graph (shown as a straight line in Figure 4) is the Seebeck coefficient , which resulted in being *S* = 0.160 mV/K. Similar graphs have been obtained for the 6.5 and 24 μm long SiNW forests, achieving very close values for *S*: 0.154 mV/K (6.5 μm) and 0.179 mV/K (24 μm). The substrate is *p*-doped with a resistivity of ρ = 0.003 Ω·m, which corresponds to a doping concentration *p* of about 3 × 10^19^ cm^–3^. It is very difficult to measure the final effective doping of the SiNWs, because the surface states, together with the high surface-to-volume ratio, can significantly modify the final hole concentration in the core of the nanowires. However, the value *S* = 0.16 mV/K is in line with that measured on bulk, heavily doped silicon.^1,37,38^

Current–voltage (*I*–*V*) characteristics (see the Supporting Information for a typical characteristic) showed a linear *I*–*V* behavior; therefore, we can claim that electrical contacts do not introduce a barrier effect. The internal resistance *R*_G_ of the *p*^+^-leg thermoelectric generator has been determined through a linear fit of the four-contact *I*–*V* characteristics. *R*_G_ resulted in the range of 3 × 10^–3^ to 20 × 10^–3^ Ω cm^2^, which is very small in absolute but quite high if compared with the resistance due to the nominal resistivity of the wafer. It is very difficult to determine the actual resistance of the nanowires themselves, because of the contact electrical resistances (see the Supporting Information for further details). Thus, the measured resistance should be considered as the entire device electrical resistance, while the resistivity of the nanowires remains unknown at this stage.

### Power Output

**Figure 5:**
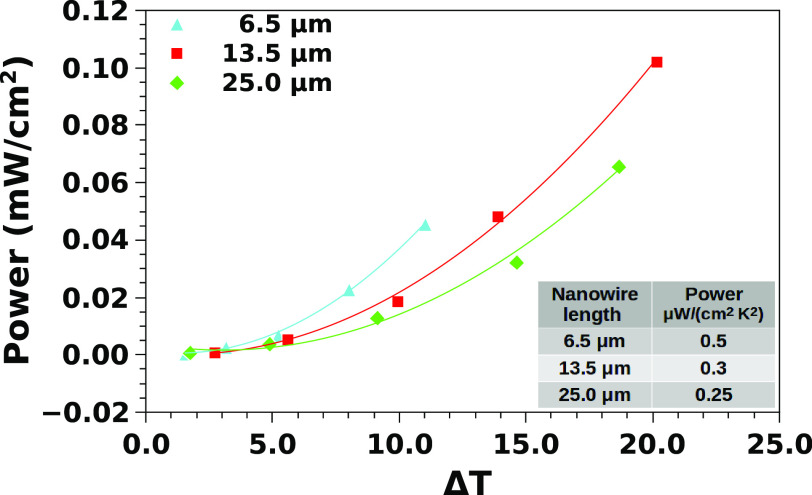
Maximum power output for a typical *p*^+^ TEG, made by a 2 h MACE etching of a silicon wafer with a nominal resistivity of 0.003 Ω cm.

As it is, a contacted *p*^+^ forest can be used as a one-leg thermoelectric generator, following the scheme shown in Figure 1b. We determined the electrical power that Si-*p*^+^ TEGs can deliver to an applied electrical load *R*_L_. To this end, we imposed a temperature difference by using the guarded hot plate setup, and we measured the open circuit voltage *V*_S_ = *S*Δ*T*. Following the well-known maximum power transfer theorem, the maximum deliverable power is achieved with an output load resistance *R*_L_ that matches the internal resistance *R*_G_ of the generator. It is straightforward that, with this loading condition, the output voltage is *V*_S_/2. The external electrical load has been applied by a Source-Meter Unit (SMU) (Keithley 2602), which has been programmed to maintain a fixed voltage *V*_S_/2 to the *p*^+^ leg. The output current resulted in being *I*_out_ = *V*_G_/2*R*_G_ in all of the measured samples. With these loading conditions, the output electrical power  is the maximum one that the TEG can generate with the given temperature difference imposed by the heat sources. Figure 5 reports the maximum output power (per cm^2^) of a TEG based on *p*^+^ nanowires of different lengths, as a function of the temperature difference. As expected, the power increases quadratically with the temperature difference, since the Seebeck voltage is proportional to the temperature difference and the power output depends on the square of the generated voltage. The table in the inset shows the electrical power output density, divided by the square of the temperature difference, as a function of the nanowire length. The output power density, divided by the square of the temperature difference, is very close to 1 μW/(cm^2^ K^2^). This is an excellent value, considering that it has been achieved with an all-silicon macroscopic, nanostructured, thermoelectric generator.

## Discussion and Conclusions

The proposed process for the fabrication of a single *p*^+^-leg thermoelectric module can be easily implemented in an industrial fabrication line for large scale production. The solution based on two legs, respectively, *p*^+^- and *n*^+^-doped, would be desirable but, unfortunately, the proved difficulty in the fabrication of long and narrow *n*^+^ nanowires precludes its application. The power output density (see table in Figure 5) is high enough for many practical applications of energy scavenging, which could benefit from the use of a low cost and sustainable material, such as silicon. Figure 5 shows that a single *p*^+^ leg can generate on the order of hundreds of μW/cm^2^ with a difference of temperature of the order of 20°. A power output of several mW/cm^2^ can be achieved with temperature differences above 100°, assuming that the power factor *S*^2^σ is constant with temperature. However, this is an underestimated value, because another advantage of silicon is that its power factor increases with temperature,^2,3,38,39^ up to temperatures of several hundreds of degrees centigrade.

The most relevant point is the very low thermal conductivity of our SiNW forests, which is 1.8 ± 0.3 W/m K. Several works reported a low thermal conductivity, measured on single silicon nanowires.^8,13,25,40,41^ In particular, nanowires fabricated by the top-down approach and smoothed by thermal oxidation showed a thermal conductivity over 10 W/(m K).^10^ A thermal conductivity smaller than 10 W/(m K) has been measured on vertical nanowire arrays fabricated by lithography and DRIE^22,23,42^ (9 W/(m K),^23^ 7.5 W/(m K),^22^ and 10.1 W(m K)^42^). In these cases, the reduction of the thermal conductivity has been ascribed to the roughness resulting from the plasma etching process.^42^ Alternatively, both VLS and MACE processes can produce large amounts of nanowires without expensive high resolution lithography. VLS produces SiNW with enhanced thermoelectric properties (good Seebeck coefficient and electrical conductivity), with thermal conductivities around 20 W/(m K),^13,14^ 18 W/(m K),^13^ and 22 W/(m K)^25^. The thermal conductivity of SiNW produced by MACE, measured on a single nanowire,^11,24,41^ resulted in being smaller than that of VLS nanowires and comprised between 4 and 5 W/(m K). This value is consistent with that reported in our previous work^36^ (*k*_t_ = 4.7 W/(m K)), measured on a slightly doped SiNW forest (macroscopic sample). The smaller thermal conductivity of MACE nanowires, with respect to the VLS or DRIE ones, can be explained considering that MACE gives very rough nanowires. It has been demonstrated, both theoretically^43^ and experimentally,^11,12^ that surface roughness is extremely effective in the reduction of the thermal conductivity. A further reduction of thermal conductivity to values around 1 W/(m K) has been demonstrated in porous nanowires:^44^*k*_t_ of 1.6 W/(m K) has been measured on porous SiNW arrays.^45^ Our *p*^+^ SiNW forests are mainly monocrystalline, but the surface roughness/surface porosity gives a very small thermal conductivity. This is fundamental for practical applications, because it will allow a significant temperature drop between its extremities.

The parameter that in our case must be improved is the electrical resistance *R*_G_. Taking into account the Seebeck coefficient (0.16 mV/K) and the measured electrical and thermal resistances, *ZT* is about 0.8 × 10^–3^ at room temperature, hence well below expectations. However, considering the high doping value and the principally crystalline core of the nanowires, the electrical resistance should turn out to be very low. If the nominal resistivity of the wafer could be used to estimate *ZT*, a value of 0.15 would be achieved. Anyhow, the parasitic electrical resistances of the substrate and of the mechanical assembly (see the Supporting Information), even if very small (tens of mΩ), prevent the precise measurement of the nanowire electrical resistivity.

As they are, thanks to the high temperature differences allowed by the reduced thermal conductivity, the single-leg *p*^+^ SiNW thermoelectric generators can be implemented in all those applications where electrical power density is more important than efficiency. Future work will focus on the development of a suitable mechanical assembly for the thermoelectric module that should provide a reduced parasitic electrical resistance. At the same time, it should allow the thinning of the substrate that, even if heavily doped, remains thick with respect to the nanowire length and, hence, still determines a resistance several times higher than that of the nanowires.

## Supporting Information Available

Details on the uni-leg TEG fabrication process applied to the differently doped silicon substrates, description of the thermal and electrical measurement techniques performed on these devices, and four contact IV characterization and discussion of parasitic resistances (ZIP)
