# Supplementary material for: High Power Thermoelectric Generator Based on Vertical Silicon Nanowires
Source: Nano Lett. 2020 May 28;20(7):4748–53. doi: 10.1021/acs.nanolett.0c00227 (PMC8007127; doi:10.1021/acs.nanolett.0c00227)
Supplement: Supplementary file 1 — nl0c00227_si_001.pdf [file nl0c00227_si_001.pdf]

High power thermoelectric generator based on vertical silicon nanowires: SUPPLEMENTARY INFORMATION

E-mail:

Nanowire forest fabrication through MACE

The nanowire forests result from the application of the Metal Assisted Chemical Etching (MACE). Silicon chips have been cleaved from standard electronic grade silicon wafers (100) oriented. The etching has then been performed plunging silicon chips of roughly 1 cm^2 in a solution containing $AgNO_3$ 0.1 N, HF 48% and deionized water. Several reagent concentrations and temperatures have been tested on: both n and p slightly doped chips, achieved by wafers with nominal resistivity 1-10 Ω·cm (doping concentration of about 10^{15} cm^{-3}); moderately doped n chips, resistivity 0.1-0.5 Ω·cm (doping concentration 10^{17} cm^{-3}); p^+ chips, resistivity 0.003 Ω·cm (doping concentration 3×10^{19} cm^{-3}); n^+ chips, resistivity 0.005 Ω·cm (doping concentration 10^{19} cm^{-3}).

When the samples are removed from the solution, a thick layer made of dendritic silver and silver nanoparticles covers the entire surface. Therefore, the chips have been cleaned in a solution of HNO_3:H_2O 1:1 for 1 minute for the removal of silver, rinsed with deionized water and blow dried with pure nitrogen. After the removal of the silver nanoparticle layer, an extensive morphological characterization has been performed by SEM inspection, both in top vision and in cross-section. The etching of the nanowire forests is determined by the [HF]/[$AgNO_3$] concentration ratio and the etching temperature. The latter is a very critical

parameter: the temperature has been maintained stable withing ± 0.5 degree by means of a cooled thermostatic bath. For low values of [HF]/[$AgNO_3$] the hole injection ratio, which is determined by the silver nitrate, is higher than the HF etching rate. Hence, holes have time to diffuse on the surface, and silicon results uniformly etched (polishing) or porous. However, for slightly doped silicon (both n and p type) there is a large range of [HF]/[$AgNO_3$] concentration ratios[1] which results in nanowire forests with high length to diameter aspect ratio. The nanowire length depends on the etching time; however, during the etching, the solution is depleted of $AgNO_3$ because silver is deposited on the top of the sample, and hence the etching rate decreases overtime because the $AgNO_3$ concentration decreases. Figure 1.a of the article shows a SEM cross-section image of a typical nanowire forest fabricated on an n-doped chip 1x1 cm^2 (nominal resistivity 1-10 Ω·cm). The etching has been performed for 8 h in a solution 5:16:60 $AgNO_3$(0.1 N):HF (48%):H_2O, at a temperature of 18 ^oC. The volume of the solution was 81 ml. After 4 h of etching, the solution has been replaced with a fresh one: the chip has been removed from the old solution, and resulted covered by a large amount of silver; then, it has been immediately soaked in the fresh solution without drying or removing the deposited Ag. We suppose that there is no limit to the etching depth (which gives the nanowire length), if a periodical replacement of the etching solution and/or a larger ratio between solution volume and sample surface are provided.

When applied to n^+ chips, the etching resulted always in porous nanostructures for all the tested etching parameters (reagent concentrations, etching temperature and time). The high etching rate resulted in long nanowires, which easily collapsed and bundled together.[2] After the removal of the deposited Ag, all the n^+ chips showed an uniform brown color to the eye, very different from that of the n (or p) chips that showed always a dark black color. The different color is due to the formation of porous silicon, which has a lower refractive index with respect to crystalline silicon.[3] The porosity of n^+ SiNW forests has been confirmed by SEM inspection: Fig. 1.b of the article shows the cross section of a typical nanowire forest on a n^+ chip, etched in a solution 5:16:60 $AgNO_3$(0.1 N):HF (48%):H_2O for 2 h at a temperature

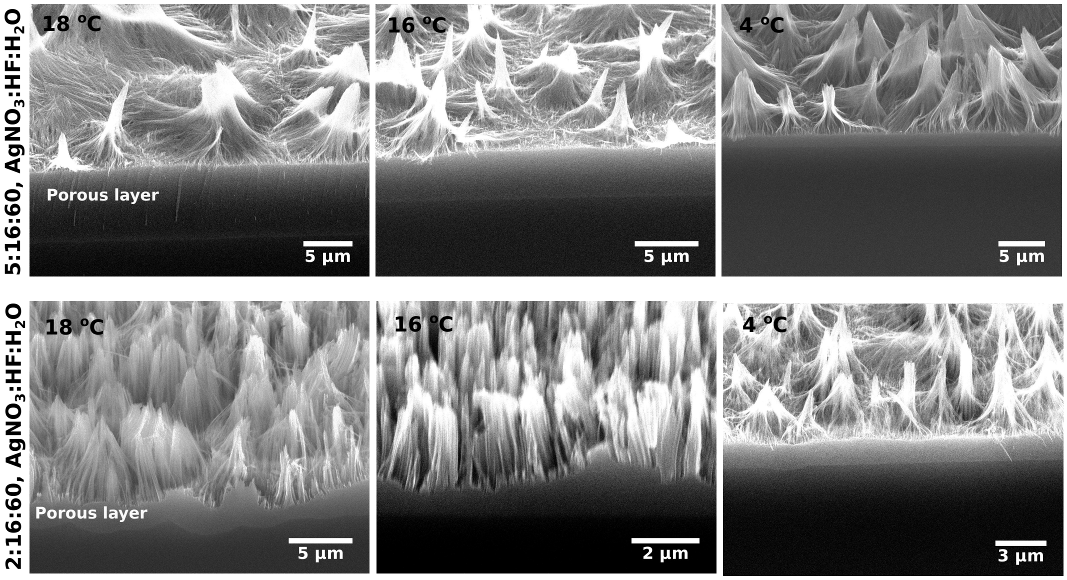

Figure 1: Silicon nanowire forests prepared on n^+ substrates with different temperatures and reagent concentrations. In all the cross-section SEM images a porous layer is visible at the bottom of the nanowires.

of 18 ^{o}C : a thick layer of porous silicon is visible in the substrate under the nanowire forest. The SEM images shown in Figure S1 compare typical results of MACE on n^+ chips, for different reagent concentrations (top row 5:16:60, bottom row 2:16:60 $AgNO_3$(0.1 N):HF(48 %):H_2O) and etching temperatures (18 ^{o}C, 12 ^{o}C, 4 ^{o}C). As expected, the thickness of the porous layer decreases with etching temperature and with the decreasing of $AgNO_3$ concentration. This confirms that the hole injection, driven by the reduction of the silver nitrate, causes the porosity.

In the case of p^+ substrates, we found a suitable combination of reagent concentrations and etching temperature, which can result in samples that, after silver removal, appeared dark black at the eye. SEM images confirm that there is no any layer of porous silicon at the bottom of the nanowires. The image of Fig. 1.c of the article has been obtained with 3h etching in a solution 3:16:60 $AgNO_3$(0.1 N):HF (48%):H_2O at a temperature of 18 ^{o}C.

Single-leg thermoelectric generators based on nanowire forests have been fabricated with p^+ single polished Si chips. The rough face of the chips has been protected during the etching, to prevent the formation of nanowires on this side (bottom side). Hence, nanowire forests

are defined by MACE perpendicular to the polished face of the chip (top side).

To complete the thermoelectric leg, a metal contact on top of the forest has been provided, as it was reported in our previous work,[4] and here briefly described. Two process steps are necessary. At first, a metal seed made of 15 nm Cr and 50 nm Cu is deposited by thermal evaporation on the top of the nanowire tips. The seed is crucial to achieve a good adhesion for the next metal layer. Then, the electrodeposition of copper is performed to obtain a 2 μm thick layer on the tips of the entire forest. The electrolytic solution composition is [$CuSO_4$]:[H_2SO_4] 0.4 M, and the electrolytic cell configuration allows only the nanowire forest to be in contact with the solution. This process has been proved to be reliable and robust, so that the growth of copper proceeds only on the top of the forest avoiding any short circuit with the substrate underneath.

The Si chips with the p^+ SiNW on the top are cleaved in pieces of roughly 2×5 mm^2, and positioned in the measurement set-up used both for the thermal and electrical characterization.

Measurement of the thermal conductivity

The characterization of the thermoelectric properties of the nanowire forest has been performed by means of a homemade set-up.[5] The system, reported in the sketch of Figure 2, is based on the guarded hot plate concept. The sample is squeezed, at a constant pressure, between a hot, T_H, and a cold, T_C, aluminum plates. The hot temperature T_H is settled through a resistive heater applied to the T_H plate. A third aluminium plate T_{HRef} is located above T_H and maintained at the same temperature T_H by means of a second resistive heater, electronically controlled in feedback. In this way, all the electrical power applied to the T_H heater is converted into thermal power ϕ, which flows only from T_H, through the sample, to T_C. All the measurements have been performed in vacuum and a radiation shield (not shown in the sketch) has been also applied and maintained at T_{HRef}, to reduce the possi-

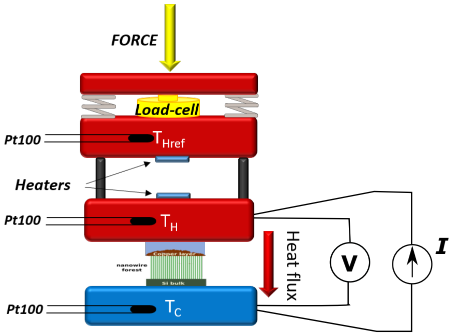

Figure 2: Sketch of the measurement system, based on the guarder hot plate technique. The electrical power, which supplies the top hot plate T$_H$, is the thermal power flowing through the sample.

ble convection and irradiation losses. The temperatures T$_H$, T$_C$ and T$_{HRef}$ are measured through Pt100 sensors, placed on the lateral sides of the plates. Moreover, as explained in detail in our previous work,[5] a Finite Element model to quantify the exact ΔT across the sample has been developed in order to take into account the temperature drop from the point in which the sensors are located to the exact contact between the plates and the sample. The measurements have been performed in steady-state conditions: once imposed a voltage V_H to the resistive heater for T_H, the system controls the heater for T_{Href} to maintain the difference $T_H - T_{Href}$ very small (below 0.1 K). A considerable amount of time (roughly 1 h) has been waited to reach the thermal stability. Once the system is stable in temperature, T_H, T_C and the current I_H of the resistive T_H heater have been recorded, so that $\Delta T = T_H - T_C$ and the thermal power ϕ=Electrical power=$V_H I_H$ have been determined. The measurement has been repeated for several values of $\phi = V_H I_H$ on each sample. Figure S3 shows measured values of ΔT as a function of the thermal power ϕ for a sample prepared with 2h of MACE on a p^+ substrate (nanowire length 13.5 μm). In the figure is also shown a linear fit of the data, which gives the thermal resistance$R_T = 9.20 \pm 0.25$ K/W. The deviation (0.25 K/W) is the standard deviation determined in the fitting procedure. Once achieved R_T for several samples, made of nanowire forests with different length L, the product R_T S has been plotted as a function of the nanowire length. S is the area of the sample (chip). A precise geometrical measurement of each chip has been performed both with a caliper and with a

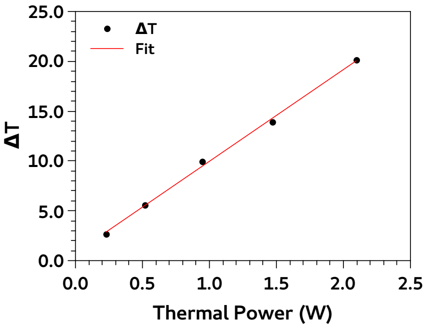

Figure 3: Temperature difference, measured as a function of the thermal power ϕ flowing through the sample, which is equivalent to the electrical power VI applied to the T_H heater.

calibrated optical microscope used at low enlargement. Hence, the surface S of each sample has been calculated. Let us indicate with R_C the contact thermal resistance of each sample. We can assume that the product $R_C\ S$, which is the contact thermal resistance per surface unit, is the same for all the samples. Hence, R_T can be expressed as:

$$R_T\ S = R_C\ S + \frac{1}{\nu k_t} L \tag{1}$$

where ν is a coverage factor; the thermal resistance of the nanowire forest can be written as $R_{NW} S = \frac{1}{nuk_t} L$. Hence, the fitting of the plot of $R_T\ S$ as a function of L gives both nuk_t and the contact thermal resistance per surface unit $R_C\ S$. The coverage factor ν is

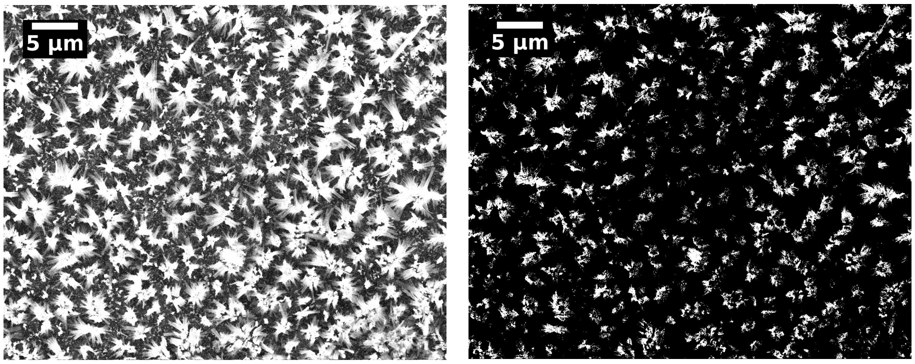

Figure 4: Left panel: a SEM image of a typical nanowire forest (2h etch on p^+ chip). The nanowires, which are perpendicular to the substrate, are bent at the top. Right panel: a software for image analysis (ImageJ) has been used for setting a threshold to show mainly the top of the nanowires; this image gives an estimation of ν.

the ratio between the effective silicon cross section area of the nanowire forest and the total area S of each sample. The procedure that we followed to estimate this parameter, which we summarize here for clarity, is reported in detail in a previous work.[5] Several planar views of each nanowire forest have been taken by SEM imaging: see for example the left panel of Figure S4. A software for image analysis (ImageJ) has been used to set a color threshold, aiming to highlight only the apexes of the nanowires (see for example the right panel of Figure S4). The area of the white part, divided for the total area of the image, gives an estimation of the coverage factor ν. This procedure has been repeated for several images taken on the same sample (at least ten images each sample), at different magnifications (between 500X and 20000X). Similar values have been obtained between different images taken on the same sample, and between different samples. The average value resulted 0.14, with a very small spread between 0.13 and 0.15.

The same experimental set-up has also been used for the measurement of the Seebeck coefficient and of the electrical characteristics. An electrical insulation under the cold plate T_C has been inserted to avoid any short circuit with the bottom part of the experimental set-up. Once reached the thermal stability of the system, the potential drop (Seebeck voltage) between the T_H and T_C blocks has been measured. The linear fit of the Seebeck voltage with respect to ΔT, as shown in Figure 4 of the main article, gives the Seebeck coefficient.

Electrical measurements

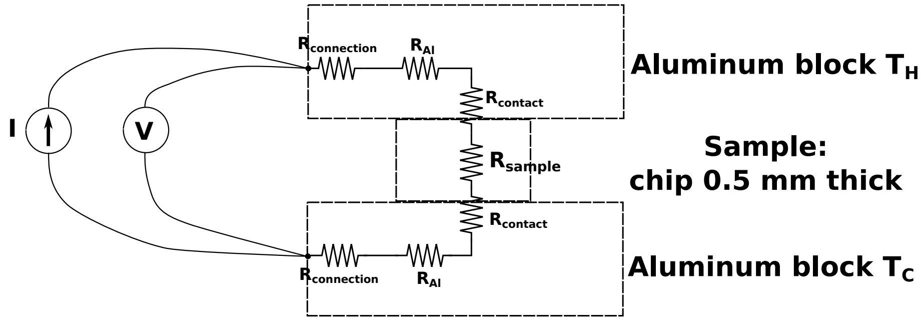

Figure 5: sketch of the parasitic electrical resistances of the measurement system.

The electrical characterization (electrical resistivity and Seebeck coefficient) has been performed in the same measurement set-up used for the thermal characterization shown in Figure S2. It must be noted that our choice to use the same measurement system both for thermal and electrical characterizations allows to measure the electrical power output in a realistic system that needs both electrical leads and plates for transferring the heat flux to the chip. Unfortunately, this choice has an important drawback related to the parasitic electrical resistances of the system that, even if very low, are not negligible with respect to that of the highly doped samples. As previously explained, the top of the nanowire forest (top of the chip) is covered by a layer of rough copper, which acts as a good contact for the silicon nanowires; the rough face of the chip (at the bottom) is covered by a copper layer deposited by thermal evaporation. As shown in the sketch, the electrical resistance of the sample is measured in four contact configuration: two contacts are used to impose a current, meanwhile the voltage is measured through other two contacts by means of a high impedance voltmeter. A picoammeter Keithley 6487 has been used for biasing and measuring the current. A high precision multimeter (6 1/2 digit) with high input impedance ($>$ 10 GΩ) (Keithley 2000) has been used for the measurement of the voltage. Two electrical connections are applied on a side of the T_H Al block at the top, the other two are applied to the bottom T_C Al block, as shown in the sketch of Fig. S2. It should be underlined that the four contact configuration avoids the resistance of the cables, but not the parasitic resistances due to the connections of the cables to the aluminum blocks; in particular, it does not avoid the parasitic contact resistances due to the aluminum-top SiNW (electrodeposited copper layer) interface, and due to the interface between the bottom of the wafer and the T_C Al block. The electrical circuit shown in Figure S5 represents the parasitic electrical resistances of the overall system. Figure S6 shows a typical $I-V$ characteristic measured in four contact configuration: the current, imposed through one couple of contacts, is reported as a function of the voltage, measured between the other two couple of contacts. Fig. S6 is related to the p^+ SiNW forest achieved with 2 h etching (length of the nanowires 13 μm , surface of the

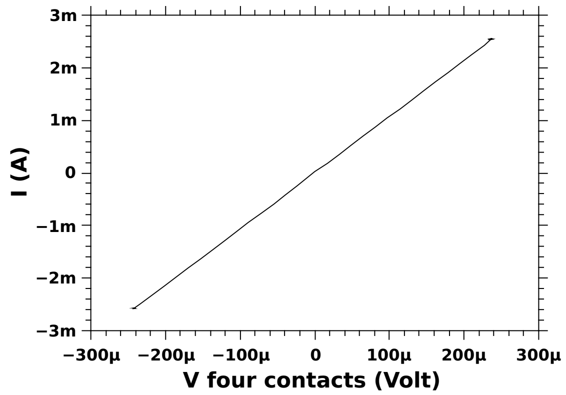

Figure 6: Typical $I - V$ characteristic, recorded in four contact configuration. The current, flowing between two top-to-bottom contacts, is reported as a function of the voltage, measured between the other couple of contacts.

chip $S = 7.7$ mm^2). The characteristic is perfectly linear, and this indicates that there are no barrier effects. In particular, this very linear characteristic demonstrates that the contact between electrodeposited copper and top of the SiNWs has a linear (Ohmic) behavior. The electrical resistance can be determined by a linear fit of this four-contact $I-V$ characteristic. For the case shown in Fig. S6, the linear fit gives a resistance of 0.093 Ω, which is very small, but quite big if compared with the nominal resistance of the samples (few milliohms, for a 10 mm^2 chip, 0.5 mm thick, with $\rho = 0.003$ Ω·cm). Indeed, it is also very high in comparison with the resistance that should result from the nominal doping of the nanowire forest with a predominantly monocrystalline core. As the electrical resistances are very small, it is reasonable that the parasitic resistances of the system, even if small, have a strong weight in the measurement. In particular, the contact resistance due to the interfaces between different materials, such as aluminum and copper (on silicon), can be very unreliable and unrepeatable. Hence, the measured resistances, which are all in the range of few tens of mΩ, cannot be ascribed to the SiNW forests. Conversely, in the case of thermal conductivity measurements, the contacts show thermal resistances which are comparable with that of the samples that are thermally very resistive. Hence, a clear trend as a function of the nanowire length can be measured, because the thermal resistance of the sample predominates with

respect to that of the contacts and of the system.

It must be noted also that the measured Seebeck coefficient (see the main text) is in reasonable agreement with the nominal doping of the wafer, which is 3×10^{19} cm^{-3} with a resistivity of 0.003 Ω·cm. The Seebeck voltage (see Figure 4 of the main article) has been measured, as a function of the temperature difference, through a high impedance voltmeter (nanovoltmenter Keithley 2182), and hence it is not affected by the parasitic electrical resistances of the system, which are very small.

References

(1) Dimaggio, E.; Narducci, D.; Pennelli, G. Fabrication of Silicon Nanowire Forests for Thermoelectric Applications by Metal-Assisted Chemical Etching. *Journal of Materials Engineering and Performance* **2018**, *27*, 6279–6285.

(2) Qi, Y.; Zhang, M.; Yang, F.; Wang, X. Processing Window for Fabricating Heavily Doped Silicon Nanowires by Metal-Assisted Chemical Etching. *J. Phys. Chem. C* **2013**, *117*, 2509025096.

(3) Chartier, C.; Bastide, S.; Levy-Clement, C. Processing Window for Fabricating Heavily Doped Silicon Nanowires by Metal-Assisted Chemical Etching. *Electrochimica Acta* **2008**, *53*, 55095516.

(4) Dimaggio, E.; Pennelli, G. Reliable Fabrication of Metal Contacts on Silicon Nanowire Forests. *Nano Letters* **2016**, *7*, 4348.

(5) Pennelli, G.; Elyamny, S.; Dimaggio, E. Thermal conductivity of silicon nanowire forests. *Nanotechnology* **2018**, *2018*, 505402.
